# Study on the impact of digital transformation on the innovation potential based on evidence from Chinese listed companies

**DOI:** 10.1038/s41598-024-56345-2

**Published:** 2024-03-14

**Authors:** Xu Zhao, Qi-an Chen, Xiaoshu Yuan, Yannan Yu, Haitao Zhang

**Affiliations:** 1 School of Economics and Business Administration, Chongqing University, Chongqing, 400030 China; 2 Surrey International Institute, Dongbei University of Finance and Economics, Dalian, 116025 Liaoning China; 3 Cesar Ritz Colleges Switzerland, English Gruss Strasse 43, 3902 Brig, Switzerland

**Keywords:** Digital transformation, Agency costs, Risk-bearing level, Enterprise innovation capability, Information technology, Socioeconomic scenarios, Sustainability

## Abstract

Digital transformation has emerged as a powerful force in reshaping the business landscape and enabling organizations to enhance their capabilities. One critical aspect of this change is how it impacts an enterprise’s innovation ability. To explore this question, we select data regarding China’s A-share listed enterprises from 2007 to 2021 as the research sample. We employ crawler technology to gather keywords related to “digital transformation” from annual reports, portraying detailed journeys of enterprises’ digital transformation. Through descriptive statistics and multiple covariance tests, a linear relationship is established between digital transformation and innovation ability. Benchmark regression is conducted and a robustness test is utilized to determine the robustness of the benchmark regression. The mechanism, heterogeneity, and moderating effects of this study are also tested. The results reveal that digital transformation makes a significant positive contribution to the innovation capability of enterprises. Meanwhile, among different types of enterprises, the impact of digital transformation on enterprise innovation capability shows heterogeneity. In terms of the impact mechanism, digital transformation can enhance the innovation output of enterprises by reducing the agency cost and improving the risk-taking level of enterprises, so as to further improve the innovation capability of enterprises. The research results of this paper provide essential theoretical support for the digital transformation of enterprises and the government’s formulation of enterprises’ digitalization strategies. More profoundly, it provides significant reference for how to further promote the digital transformation of Chinese enterprises.

## Introduction

With the integration of digital technology and the real economy, the digital economy is gradually replacing the traditional economy as a new driving force for global economic development. According to Xu and Zhang^1^, the explosive development and application of artificial intelligence, blockchain, cloud computing, and big data in recent years have enabled data to gradually become a new engine driving economic development in addition to land, labor, and capital factors. The report of the 19th National Congress of the Communist Party of China points out the need to “promote the deep integration of the internet, big data, artificial intelligence, and the real economy” and build a “digital China”. The report of the 20th National Congress of the Communist Party of China further clarifies the need to “accelerate the development of the digital economy and promote the deep integration of the digital economy and the real economy”. Since the outbreak of the bilateral trade conflict between China and the US in 2018, downward pressure on China’s economy has increased, coupled with a sharp rise in external input risks. Some export-oriented enterprises have also been greatly influenced by this economic and trade friction, and the impacts of this war are still lingering. In addition, the COVID-19 pandemic that broke out in 2020 has had a significant impact on the global economy that cannot be ignored. During this period, the application of the digital economy has enabled enterprises to gradually improve epidemic prevention and control, realize the resumption of production, and inject new impetus into the revival of China and even the global economy. Therefore, driven by the dual factors of existing policies and the above backdrop, enterprises actively try to turn crises into opportunities. Yang and Liu^2^ point that, taking digital transformation as a form of breakthrough, enterprises seek opportunities to complete innovation and remodeling in their harsh living environments, and vigorously promote the epoch-making process of the digital economy and digital transformation’s reform and innovation.

According to the report “White Paper on the Development of China’s Digital Economy”^3^, the scale of China’s digital economy reached 45.5 trillion yuan in 2021, with a year-on-year nominal growth of 16.2%. The structure of the digital economy has also been further optimized. The human economy and society itself have officially entered a new era, with “data” as its core^4^. The development environment of enterprises has been significantly subverted. Moreover, the process and results of their digital transformation have become a hot issue in political studies. In essence, the digital economy can be divided into digital industrialization and industrial digitalization. One of the core components of industrial digitalization is the digital transformation of enterprises. Digital transformation reflects the transformation process involved in an enterprise’s movement from a traditional to a digital management mode. Therefore, the introduction of new digital technology has led to the macro and systematic evolution of the enterprise at the micro level of competition, business model, operation process, and even business ecology^5^, driving the enterprise’s resource allocation in the direction of intelligence, precision, and efficiency, while simultaneously empowering the innovation performance and value of enterprises^6^.

Meanwhile, as the integration of the real economy and digital technology deepen, the digital economy is gradually replacing the traditional economy as the new engine of global economic development. The integration of enterprises and digital technology reflects the process by which enterprises move from tradition to digitization—the digital transformation. Innovation has always been a key element throughout this transformation process. The concept of digital transformation was proposed by Negroponte^7^, who viewed enterprises’ digital transformation as the digital penetration of production factors, the digital restructuring of production relationships, and the digital innovation of business activities. Some scholars have suggested that enterprises’ digital transformation is a process of transforming production processes and organizational structures through digital technology^8^. Based on this, Frynas, Mol and Mellahi^5^ provided an explanation of digital transformation, which states that the introduction of digital technology leads to a systematic evolution across the concepts of competition, business models, operational processes, and even business ecosystems, driving resource allocation in enterprises toward intelligent, precise, and efficient directions. Therefore, Wei, Gong and Liu^9^ summarized enterprise digital transformation as the advanced transformation involved in utilizing a new generation of information technology to change and upgrade existing technological and production systems, in order to optimize production methods and improve management levels.

Scholars have mainly focused on three elements when studying the impact of digital transformation on enterprise innovation capabilities: technological innovation, institutional innovation, and management innovation. First, in terms of technological innovation, enterprises’ technological innovation is an explicit ability, and digitization is a process—not a single link—that can enhance innovation capabilities through new product development, process improvement, and technological application innovations^10,11^. Second, institutional innovation involves updating organizational structures, practices, and cultures, and formulating employee stock ownership plans to maximize innovation capabilities^12–14^. Finally, in terms of management innovation, personalized services are provided by big data analysis to increase customer viscosity, a flat organizational structure is constructed to strengthen inter-departmental collaboration, and enterprises’ digital division of labor promotes platform ecosystem integration^15,16^. Based on the literature above, it can be seen that research related to enterprise digital transformation and innovation capabilities mainly focuses on the manifestation of innovation capabilities and their positive effects on enterprises, but further exploration of their specific impact mechanisms is still needed. Therefore, this chapter identifies the impact mechanism of digital transformation on enterprise innovation capabilities as an important component of this field of research.

The main innovations of this research are as follows: First, previous studies on innovation capabilities have mostly used the number of patent applications as a proxy variable, which limits the innovative behavior of enterprises to a single strategic behavior^17–19^. This approach also limits the guidance and practical significance of related literature in regard to enhancing enterprises’ own innovation capabilities^20^. Therefore, this chapter applies an innovation variable group, taking cumulative acquired R&D patents as the core dependent variable and further dividing it into invention creation patents, utility model patents, and design patents. This balances the measurement of innovation capabilities while refining enterprise R&D results, providing verifiable evidence in regard to utilizing digital transformation to enhance enterprises’ own innovation capabilities. Second, to address the problem of reverse causal interference and time lag in previous research on the relationship between digital transformation and innovation capabilities, this chapter borrows from Fishman and Svensson^21^ and handles the lag of the independent variable by using the industry average value of digital transformation as an instrumental variable for 2SLS estimation, effectively weakening the endogeneity problem. Third, based on the verification of the impact of digital transformation on enterprise innovation capability, this chapter discusses its impact mechanism from the perspectives of agency cost and risk-bearing level. Finally, in-depth research on the impact of digital transformation on enterprise innovation capabilities has practical guidance and inspirational significance for enterprise operators and policy makers. On the one hand, it helps enterprise operators better understand and promote their own digital transformation, especially by selecting appropriate risk-bearing levels according to their own development needs, thereby effectively enhancing their innovation capabilities. On the other hand, to maximize the enhancement of innovation capabilities through digital transformation, policymakers should not only focus on the enterprise’s own technological strength but also fully leverage the role of the capital market to guide and encourage enterprises to improve their innovation capabilities.

## Method

### Theoretical analysis and hypothesis formulation

#### The impact of digital transformation on enterprises’ innovation capability

The advent of the digital economy has led to the emergence of numerous new enterprises in the market. Digital transformation has become a necessary path for existing enterprises to seek breakthroughs. In order to achieve sustainable development, enterprises must constantly evolve and transform. Digital transformation can help optimize operational processes, enrich business models, and reshape organizational structures, thereby enabling enterprises to achieve self-innovation in multiple respects^22^. Therefore, an increasing number of enterprises are aiming to become “digital enterprises” in order to accelerate their transformation and unleash their innovative potential. Among them, the introduction of advanced digital technologies is one of the main features of digital transformation. An^22^ believes that the essence of digital transformation lies in the utilization of digital technologies to solve complex and uncertain problems, thereby enhancing innovation capability and operational efficiency. Vial^10^ point out that the application of digital technologies can help enterprises achieve improvements and optimization in various aspects. Based on the above research, this study defines digital transformation as the change and upgrade of corporate governance at the enterprise level due to the integration of digital technologies, with the goal of achieving the rational utilization of resources in daily operations.

Enterprise innovation is a continuous economic process. Schumpeter^23^ first mentioned in his book, *The Theory of Economic Development*, that innovation needs to be improved in various respects, such as procurement models, production methods, organizational structures, and research and development. Therefore, the forms innovation can take are diverse, with the most significant being innovation output. Research and development activities are the foundation and core of enterprise innovation output, and digital transformation provides new methods and ideas for enterprise research and development activities. By analyzing and processing resources and data information through digital technology, enterprises can optimize the research and development process and match various target tasks with the best resources and talents^24^, thereby achieving optimal outputs at each stage of the research and development activities and improving their innovation capability. Most existing studies regard the number of patent applications or research and development investments as indicators of enterprise innovation capability^20,25,26^. However, different types of patents actually reflect different levels of enterprise innovation capability, and there are also significant risks in transforming research and development investment into innovation capability. This study enriches the measurement of innovation capability by classifying the output of innovation into three categories according to the types of patent applications in China.

According to resource-based theory, the integration and allocation of resources are important factors for the success of enterprise innovation, including internal and external factors. From an internal perspective, Loebbecke and Picot^27^ believe that, with the deepening of digital transformation, the operational efficiency of enterprises will be greatly improved. Digital technology enables enterprises to improve resource utilization at lower costs, thereby enabling enterprises to achieve higher outputs under existing innovation resource conditions. Li, Dang and Zhao^28^ point out that digital technology can more effectively utilize innovation resources and reduce repeated investments and resource waste in traditional enterprise operating structures. From an external perspective, enterprises can use digital technology to quickly capture target resources and other associated resources, assisting in establishing “weak ties” outside the organization^29^. Autio, Nambisan, Thomas and Wright^30^ propose that, when enterprises interact with external stakeholders, they can use digital technology to build better management systems and better platforms for efficient communication. Enterprises can acquire more advanced external knowledge through digital technology to improve product innovation speed^31^.

It is worth mentioning that the innovation capability of enterprises is also influenced by many other factors. At the macro level, industrial policies^32^, fiscal and technological investment^33^, institutional environment^34^, and capital market reforms^35^ can all bring about changes in innovation capability. At the micro level, innovation capability is often affected by factors such as the leverage levels of enterprises^36^, the characteristics of top management^37^, corporate culture^38^, and forms of corporate ownership^39^. Meanwhile, enterprise innovation capability is also influenced by many negative factors, such as weak internal control processes and high uncertainty risks^40,41^. According to the research results of Li and Wang^42^, as environmental uncertainty factors increase, the role of digital transformation in promoting innovation by enterprises will become greater. Based on the above analysis, this study posits that, the stronger the digital transformation is in enterprises, the stronger their innovation capability will be.

This paper thus proposes hypothesis H1: Digital transformation can enhance enterprise innovation capability.

#### The impact of digital transformation on enterprise innovation capability through agency costs

Traced back to its origins, the internal costs caused by the conflicts of interests between shareholders (principals) and management (agents), as well as the costs incurred in handling these differences and contradictions, are called agency costs. Foundational studies in this field suggest that there is often information asymmetry between shareholders and management teams^43^, and the consideration of moral hazards in management teams cannot be ignored^44^. The distribution of interests between shareholders and management teams is also part of corporate governance, and mishandling it can result in agency costs, including contract costs, management costs, and regulatory costs. Therefore, shareholders need to take certain measures to minimize agency costs and to supervise and control management teams to the maximum extent possible.

According to Jensen and Meckling^45^, the agency problem that exists between principal and agent is referred to as the first type of agency problem. In the process of corporate governance, it is not ideal for the principal to have complete supervision over the agent. Under conditions where the principal is at an informational disadvantage, the agent tends to act in their own interests, seeking a higher fixed wage income rather than taking on the income risk associated with innovation, uncertainty, and long-term considerations^46^. Furthermore, the second type of agency problem between large and small shareholders essentially involves the oppression of small shareholders by large shareholders, leading to high coordination costs among shareholders^47^. Given the significant risks involved in the innovation process and the need for long-term considerations, the high transaction costs of reaching a consensus between the two types of shareholders in the enterprise’s innovation decision-making process can lead to a tendency for the actual controllers of the enterprise to engage in asset stripping, thereby exacerbating the enterprise’s financing constraints^48^.

To effectively address and mitigate such agency problems, various measures have been introduced both domestically and internationally, such as the establishment of an external director system and the formation of specialized state-owned asset management institutions, but their effectiveness remains limited. Regarding the external director system, many scholars have raised doubts about whether independent directors can truly exercise regulatory functions^49^. However, with the increasing number of companies joining the wave of digital transformation, digital technology has effectively addressed issues such as information asymmetry, information transaction costs, and agency costs^50^. Principals have achieved convenient, fast, and low-cost information acquisition through digital networks. This indicates that digital technology plays a significant role in addressing information asymmetry issues. Digital technology has become a major factor in improving corporate management, leading to a downward trend in the agency costs of principals in this context. Furthermore, digital transformation is being fully integrated into the business ecosystem of enterprises, indicating that the data resources within enterprises are gradually being managed in a more refined and scientific manner^51^. Consequently, agents find it difficult to determine the direction of innovation based on personal desires, and digital technology will also promote further transparency in the enterprise management process. This approach can reduce the monitoring costs of principals in terms of management behavior and the agency costs between managers and general employees. Overall, the organizational transformation brought about by the digital transformation of enterprises has led to the effective and comprehensive penetration of digital technology in both business and functional management processes^52^.

At the same time, research findings related to agency costs and enterprises’ innovation capabilities suggest there are severe agency problems arising from conflicting interests within the enterprise, which affect the decision-making and efficiency of the enterprise’s technological innovation^53^. Peng and Luo^54^ believe that, by reducing agency costs, shareholders can obtain more information, correct the information disadvantage in competition with senior managers, and effectively suppress the pressure of the management in regard to innovative activities, thus enhancing the enterprise’s innovation performance from a long-term perspective. Tang and Zuo^55^ argue that the high agency costs in enterprises lead to enterprises’ reluctance to engage in long-term, high-risk research and development activities. Therefore, to achieve sustainable development, it is necessary to reduce the agency costs of enterprises through digital transformation, thereby enhancing enterprises’ innovation capabilities.

This paper thus proposes hypothesis H2a: The digital transformation of enterprises can reduce agency costs and thereby enhance the innovation capabilities of enterprises.

#### The impact of digital transformation on enterprise innovation capability through risk-bearing level

Risk-bearing level refers to the voluntary assumption of risks by enterprises, whether rational or irrational, which manifest in the tendency of enterprises to bear daily operational costs in exchange for substantial profits^56^. The specific level of risk-bearing mainly reflects the degree of operational and financial risk undertaken by enterprises. In the daily operations and investment activities of enterprise digital transformation, risk is a key factor determining whether internal economic activities can proceed smoothly. In terms of the innovation capabilities of an enterprise, the uncertainty of risk-bearing level can hinder the smooth conduct of innovation activities, thus imposing higher demands on an enterprise’s risk-bearing level for its own innovation activities.

According to resource-based theory, first, new technologies such as artificial intelligence, big data, and cloud computing are effectively utilized in the new era of digital transformation, enabling enterprises to break traditional constraints, flexibly integrate market dynamic information with their own operating conditions, continuously analyze and identify personalized consumer needs, and effectively enhance their sensitive responses to market fluctuations. New technologies also improve the efficiency of resource allocation in daily operations^57^, significantly reducing operational risks and increasing the risk-bearing level of enterprises. Second, data circulating within the enterprise are maximally developed through the application of digital technology^58^, further maximizing the effective and reliable financial data available for grasping the current development of enterprise operations, achieving the maximization of resource allocation, and enhancing the risk-bearing level of enterprises. Finally, because digital transformation is a procedural behavior, resource-rich enterprises can tolerate partial or temporary stagnation resulting from failure^59^, alleviating the impact of digital innovation uncertainty on enterprises^60^. In addition, digital transformation can mitigate the principal–agent problem and promote the learning of relevant knowledge by management. By improving the information transparency of enterprises, digital transformation enables stakeholders to better supervise managers and restrain their risk-avoidance behavior driven by personal interests. It mitigates the issues of adverse selection and moral hazards, thereby enhancing the risk-bearing level of enterprises^14,61^. Meanwhile, in the optimization and restructuring of internal and external environments, digital transformation requires managers to learn advanced technology theories and management skills^62^. Based on the theory of higher-order gradients, the cognitive level of managers influences their decision-making, and their management abilities continuously improve with the accumulation of theoretical knowledge. This enables managers to adapt to rapidly changing environments and dare to choose high-risk investment projects^63^, ultimately enhancing the risk-bearing level of enterprises.

The level of risk-bearing by enterprises affects the innovation capabilities of enterprises^64^. On the one hand, a high risk-bearing level helps enterprises to raise funds to a greater extent, providing financial support for innovative activities, enhancing the motivation and preventive mechanisms of cash holdings^65,66^, and making it easier for enterprises to fully realize the value of resources and invest them in long-term innovative projects such as research and development^67^. Second, the enhanced confidence of the management resulting from a high risk-bearing level will expand the range of choices available for the daily operations and project investments of enterprises, further driving the expansion of innovative activities within the enterprises^68,69^. On the other hand, the complexity of digital technology and the uncertainty of innovation increase the risk of transformation failure^50^. Enterprises with low risk-bearing levels urgently need stable investment and expansion, making it difficult to invest limited resources in complex innovation activities^59,60^. Based on the above studies, this paper suggests that digital transformation can enhance the innovation capabilities of enterprises by improving their risk-bearing levels.

This paper thus proposes hypothesis H2b: The digital transformation of enterprises can enhance their risk-bearing level and thereby improve the innovation capabilities of enterprises.

#### The moderating effects of corporate ownership nature, percentage of institutional investors, percentage of overseas business income, and industry concentration on the relationship between digital transformation and enterprise innovation capability

Research has found that the level of innovation capability resulting from enterprises’ digital transformation is influenced by the nature of corporate ownership^70^. However, most studies have focused on the comparison between state-owned enterprises and private enterprises, with little research on the differences between state-owned and non-state-owned enterprises. First, compared to non-state-owned enterprises, state-owned enterprises are more likely to obtain government resources and information support, breaking through funding constraints and technological barriers in the innovation and research process^49^. Second, state-owned enterprises have accumulated scientific research, talent, and technology, which can generate economies of scale in collaborative innovation with upstream and downstream companies in the industrial chain^71^, facilitating the evolution of the enterprise’s innovation ecology and the enhancement of its innovation capability. Finally, state-owned enterprises often exhibit a stronger sense of social responsibility; to better fulfill their responsibilities in ensuring employment and maintaining social stability, they are more likely to demonstrate a strong motivation for policy implementation and innovation breakthroughs^72^.

Based on the above analysis, hypothesis H3a is proposed: The effect of digital transformation on the innovation capability of state-owned enterprises is stronger than that on non-state-owned enterprises.

From the perspective of institutional investors, digital transformation is currently a popular topic in the investment field. Companies that choose digital transformation and disclose relevant information externally signal their active participation in the capital market, arousing investor interest and obtaining more business investments, thereby attracting more attention in the capital market. Jensen and Meckling^45^ argue that the long-term ownership and high exit costs associated with institutional investors make them more concerned about innovative activities that can generate returns. Most institutional investors actively participate in the supervision, management, and governance of companies, minimizing the possibility of independent decision-making errors by the corporate management and motivating them to innovate^73^. Therefore, there is a clear positive correlation between the shareholding proportion of institutional investors and an enterprise’s innovation capability^74^. However, there is currently limited research on the moderating effect of institutional investors’ shareholding on the relationship between digital transformation and enterprise innovation capability.

This paper thus proposes hypothesis H3b: Institutional investors’ shareholding proportion has a positive moderating effect on the relationship between digital transformation and enterprises’ innovation capability.

According to dynamic capability theory, the transformation and upgrading of enterprise development strategies will bring about fundamental changes in various aspects of operation and management, directly affecting the formulation and implementation of international business strategies^75^. With the expansion of overseas business and the accompanying increased level of openness, the implementation of the new strategy of digital transformation will further strengthen the dynamic capabilities of enterprises, enhancing their adaptability and innovation capabilities. In order to better enter international markets, enterprises must fully leverage the competitive ability and improved innovation capabilities brought about by digital transformation. First, to expand to overseas markets, enterprises improve factor allocation through digital transformation, enhance production efficiency^76^, and continuously increase the spillover of innovative technology. Second, to further expand their level of openness, enterprises pay more attention to digital transformation and the application of digital technology, effectively reducing the costs and risks of overseas business operations^77^, with multidimensional forms of innovation across technology, operations, and management playing a critical role.

Based on the above analysis, hypothesis H3c is proposed: An enterprise’s level of openness positively moderates the relationship between an enterprise’s digital transformation and innovation capability.

As China’s high-tech enterprises continue to grow, digital transformation is actively leading companies toward further innovation. However, enterprise innovation significantly increases its operational risks. Enterprises do not naturally prefer innovation; innovation is determined by the level of industry competition. When industry concentration is low, enterprises with lower market shares attempt to seize a greater market share through innovation^78–81^. Conversely, when industry concentration is too high, market monopolization leads to decreased competition, making it difficult for enterprises to focus on their innovation capabilities^82,83^. In the long run, larger enterprises with higher industry concentration will dominate the market. In order to obtain higher profits, these enterprises will often manipulate product prices to gain profits, overlooking research and development, resulting in a decrease in the intensity of the enterprise’s research and development activities^84^ and thereby inhibiting enterprise innovation.

Therefore, this paper proposes hypothesis H3d: Industry concentration negatively moderates the effect of digital transformation on the innovation capability of enterprises.

## Research design

### Data source

This paper selects A-share listed enterprises from 2007 to 2021 as the initial sample. It thus covers both enterprise samples before digital transformation and samples that have not yet undergone digital transformation at the sample observation time point, so as to avoid the endogenous interference caused by selective errors as far as possible and ensure the reliability of the regression results. The financial data regarding the enterprise comes from the Wind database. To eliminate the interference of certain special observation samples on the empirical results, this article processed the data as follows: (1) Exclude financial industry samples to avoid interference, such as differences in accounting standards; (2) exclude companies with an abnormal listing status, such as ST and PT, and avoid interfering with the overall regression results due to any abnormal business operations of the companies themselves; (3) eliminate observation samples with large quantities of data missing; (4) the continuous variables in the data shall be shrunk at the level of 1% from the beginning to the end to avoid interference due to extreme outliers. Based on the cumulative total number of R&D patents obtained (${{\text{ln}}\_patentcul}_{it}$), the cumulative number of invention and creation patents obtained (${{\text{ln}}\_inventioncul}_{it}$), the cumulative number of utility model patents obtained (${{\text{ln}}\_utilitymodelcul}_{it}$), and the cumulative number of design patents obtained (${{\text{ln}}\_designcul}_{it}$), the final observations are 4292, 3987, 2821, and 1752, respectively. The software used in this study is Stata 17.0.

### Variable settings

#### Dependent variables

Innovation variable group. Previous studies have mostly used the number of enterprise patent applications or R&D investment as proxy variables for enterprise innovation capability. However, in reality, different types of patents portray varying degrees of an enterprise’s innovation capability, and there is a significant risk that R&D investment will not effectively transform into innovation capability. Accordingly, this article takes the cumulative total number of R&D patents obtained by the enterprise (${{\text{ln}}\_patentcul}_{it}$) as the proxy variable for the innovation ability of the enterprise, and classifies patents according to different types. The first is the cumulative number of invention and creation patents obtained (${{\text{ln}}\_inventioncul}_{it}$); the second is the cumulative number of utility model patents obtained (${{\text{ln}}\_utilitymodelcul}_{it}$); and the third is the cumulative number of design patents obtained (${{\text{ln}}\_designcul}_{it}$). This approach can both eliminate the interference brought about by design patents with low technology and preliminarily test the robustness level of the results.

#### Independent variables

The independent variable in this paper is the enterprises’ digital transformation. Both the business community and the academic community have discussed how to measure the strength of enterprises’ digital transformation. Qi and Xiao^15^ believe that enterprises’ digital transformation takes “ABCD” (artificial intelligence, blockchain, cloud computing, big data) technology as the core infrastructure, and this level of digital transformation focuses on embedding digital technology into the daily operations of enterprises. Furthermore, in this way, enterprises aim to empower production technology, management, and sales through the application of digital technology. From a technical standpoint in regard to variable design, this article utilizes the Python web scraping functionality to compile annual reports of all A-share listed companies. The Java PDFbox library was employed to extract the content of these reports. Drawing on established research, the study measured the digital transformation in sampled enterprises by calculating the total frequency of five key terms: “artificial intelligence technology”, “blockchain technology”, “cloud computing technology”, “big data technology”, and “digital technology application”^4,85^. To mitigate issues such as heteroscedasticity, the study added one to the count of each term’s occurrence per year and then applied natural logarithm transformation.

#### Mediated transmission variables

The agency cost (ATO). This paper draws on the practices of Singh, Davidson and Suchard^86^, Xiao and Chen^87^, and Xu and Zhang^1^, and selects the total asset turnover (ATO) as the proxy variable to measure the enterprises agency cost. The higher the total asset turnover of ATO, the lower the enterprise’s agency cost.

Risk-bearing (Risk). Using the approach adopted by Yu, Li and Pan^88^ and Song, Wen, Wang and Shen^63^, the volatility of corporate profits (Risk) is used to measure the risk-bearing level of enterprises. The higher the value of Risk, the higher the enterprise’s earnings volatility.

#### Control variables

To overcome the impact of missing variables as much as possible, this article refers to previous literature and includes multiple variables at the micro level of the enterprise. This includes five control variables: enterprise size (Size), asset structure (Lev), profitability (ROA), proportion of independent directors (Indep), equity concentration ratio (Top10), and cash flow situation (Cashflow)^89^.

#### Moderator

Considering the way in which enterprises’ digital transformation may correspond with changes in internal and external factors, based on the literature review, this paper utilizes enterprise attributes (SOE), overseas business income proportion (Overinc), institutional investor shareholding ratio (INST), and industry concentration ratio (HHI) as moderating variables.

The specific variable definitions are detailed in Table 1.

**Table 1:** Variable definitions.

Variable type	Variable code	Variable name	Variable construction
Dependent variable	${{\text{ln}}\_patentcul}_{it}$	The cumulative total number of R&D patents	Log (1 + the cumulative total number of R&D patents)
	${{\text{ln}}\_inventioncul}_{it}$	The cumulative number of invention and creation patents	Log (1 + the cumulative number of invention and creation patents)
	${{\text{ln}}\_utilitymodelcul}_{it}$	The cumulative number of utility model patents	Log (1 + the cumulative number of utility model patents)
	${{\text{ln}}\_designcul}_{it}$	The cumulative number of design patents	Log (1 + the cumulative number of design patents)
Independent variable	${\text{ln}}\_digi2$	The intensity of enterprise’ digital transformation	Log (1 + ABCD total entries)
Mechanical variable	$ATO$	Total asset turnover	Operating income/Average total assets
	$Risk$	Enterprises’ risk-bearing	Volatility of corporate profits
Control variable	$Size$	Enterprises’ size (logarithm of total assets)	Log (total assets of the enterprise)
	$Lev$	Asset structure (asset liability ratio)	Total liabilities/total assets
	$ROA$	Profitability (return on total assets)	Net profit/average balance of total assets
	$Indep$	Proportion of independent directors	Number of independent directors/total number of directors
	$Top10$	Equity concentration ratio	Sum of the shareholding ratios of the top 10 shareholders of the enterprise
	$Cashflow$	Cash flow situation	Proportion of operating cash flow to operating income
Moderator	$HHI$	Industry concentration ratio	Herfindahl index
	$INST$	Shareholding ratio of institutional investors	The total number of shares held by institutional investors/circulating share capital
	$Overinc$	Proportion of overseas business revenue	Overseas business income/Total income
	$SOE$	Enterprise attributes	State-owned enterprises equal 1, while private enterprises equal 0

### Model setting and empirical testing

Referring to existing research methods, this article establishes the following model:

1 $$In\_patentcul_{it} = \alpha_{1} In\_digi2_{i,t - 1} + \alpha_{2} Size_{i,t} + \alpha_{3} Lev_{i,t} + \alpha_{4} ROA_{i,t} + \alpha_{5} Indep_{i,t} + \alpha_{6} Top10_{i,t} + \alpha_{7} Cashflow_{i,t} + cons + Year + Industry + \in_{it}$$

2 $$In\_inventioncul_{it} = \alpha_{1} In\_digi2_{i,t - 1} + \alpha_{2} Size_{i,t} + \alpha_{3} Lev_{i,t} + \alpha_{4} ROA_{i,t} + \alpha_{5} Indep_{i,t} + \alpha_{6} Top10_{i,t} + \alpha_{7} Cashflow_{i,t} + cons + Year + Industry + \in_{it}$$

3 $$In\_utilitymodelcul_{it} = \alpha_{1} In\_digi2_{i,t - 1} + \alpha_{2} Size_{i,t} + \alpha_{3} Lev_{i,t} + \alpha_{4} ROA_{i,t} + \alpha_{5} Indep_{i,t} + \alpha_{{6}} Top10_{i,t} + \alpha_{{7}} Cashflow_{i,t} + cons + Year + Industry + \in_{it}$$

4 $$In\_designcul_{it} = \alpha_{1} In\_digi2_{i,t - 1} + \alpha_{2} Size_{i,t} + \alpha_{3} Lev_{i,t} + \alpha_{4} ROA_{i,t} + \alpha_{5} Indep_{i,t} + \alpha_{6} Top10_{i,t} + \alpha_{7} Cashflow_{i,t} + cons + Year + Industry + \in_{it}$$

In regression Eqs. (1), (2), (3), and (4), the dependent variables are the cumulative total number of R&D patents obtained by the enterprise, invention and creation patents obtained, utility model patents obtained, and design patents obtained. The core independent variable is the enterprise’s digital transformation (${a}_{1}{{\text{ln}}\_digi2}_{i,t-1}$); the control variable consists of relevant financial indicators and operational indicators of enterprise i in year t; and ${\in }_{it}$ is a random error term. This paper carries out the following processing: First, considering that the impact of digital transformation on enterprise innovation capability may have a time lag and in order to avoid endogenous interference caused by potential reverse causality, this paper lags the core dependent variable for a period of time. Second, to absorb related fixed effects and avoid the interference of omitted variables, this article adopts both individual and time fixed effects, while considering the possibility of certain industry characteristics changing with different industries. This study also controls industry fixed effects for testing.

## Empirical results and analysis

### Descriptive statistical analysis

Table 2 presents the descriptive statistics for the main variables in this paper. The cumulative total number of R&D patents acquired by firms (${{\text{ln}}\_patentcul}_{it}$) is the main independent variable in this paper, reflecting the innovation capabilities of enterprises. To further compare the impact of digital transformation on the different types of patents in this study, the cumulative total number of R&D patents acquired is also broken down into the cumulative total number of invention patents acquired (${{\text{ln}}\_inventioncul}_{it}$), utility model patents (${{\text{ln}}\_utilitymodelcul}_{it}$), and the cumulative number of designs acquired (${{\text{ln}}\_designcul}_{it}$). The sample mean and standard deviation of the cumulative total number of R&D patents acquired (${{\text{ln}}\_patentcul}_{it}$) have a sample mean and standard deviation of 4.8559 and 1.3222, and minimum and maximum values of 0.6931 and 10.6085, respectively, indicating that the firms in the study sample of this article generally have a certain number of patents, but the patent data have a large dispersion (i.e., reflecting the differences in R&D performance among firms. The companies have patents for inventions (${{\text{ln}}\_inventioncul}_{it}$) and patents for utility models (${{\text{ln}}\_utilitymodelcul}_{it}$). The utility models (${{\text{ln}}\_designcul}_{it}$) have averages of 3.2211, 3.9675, and 2.5861, respectively, indicating that patents acquired by A-share listed companies consist mainly of patents for inventions and utility models, while patents for industrial designs and with low technology are relatively rare.

**Table 2:** Descriptive statistics.

Variables	(1)	(2)	(3)	(4)	(5)
	N	Mean	Sd	Min	Max
ln_patentscul	31,812	4.8559	1.3222	0.6931	10.6085
ln_inventioncul	31,812	3.2211	1.3919	0.0000	9.2725
ln_utilitymodelcul	31,812	3.9675	1.4390	0.0000	9.5860
ln_designcul	31,812	2.5861	1.6308	0.0000	8.5442
ln_digi2	31,812	1.1820	1.3599	0.0000	6.3008
ATO	31,812	0.6780	0.5853	− 0.0479	12.3729
Risk	31,812	1.9494	129.9206	0.0000	14,331.0524
SOE	31,823	0.4242	0.4942	0.0000	1.0000
Overinc	31,812	1.0074	4.9631	0.0000	1.4345
HHI	31,812	0.1299	0.1379	0.0142	1.0000
INST	31,812	0.3948	0.2380	0.0000	4.2682
Size	31,812	22.2116	1.3586	10.8422	28.6365
Lev	31,812	0.4859	1.6105	0.0017	178.3455
ROA	31,812	0.0351	0.4103	− 64.8192	20.7876
Indep	31,812	0.3741	0.0561	0.0000	1.0000
Top10	31,812	0.5643	0.1527	0.0131	1.0116
Cashflow	31,812	0.0469	0.0882	− 4.2696	2.2216

### Multicollinearity test

To prevent multicollinearity between the main independent variables and the control variables from affecting the accuracy of the regression results, this paper excludes multicollinearity using a variance inflation factor (VIF) test.

Table 3 shows the VIF values between the main independent variables, as well as the control variables. All values are below the threshold of five that detects the presence of multicollinearity; this test thus rules out relevant interference.

**Table 3:** VIF values for the main variables.

Variant	VIF value	Variant	VIF value
Lev	1.66	Size	1.55
ROA	1.40	Cashflow	1.26
Top10	1.08	Indep	1.01
Ln_digi2	1.03		

### Results of the benchmark regression

Columns (1) to (4) of Table 4 show the impact of enterprises’ digital transformation on the output of the above four types of patents, where the independent variables are the total number of R&D patents acquired, the total number of patents for inventions acquired, the total number of patents for utility models acquired, and the total number of patents for industrial designs acquired, respectively. Table 4 shows the results of the regression of Models (1), (2), (3), and (4) in regard to the effect of digital transformation on innovation capabilities. Columns (1) to (4) show that the estimated coefficients of the main independent variable of digital transformation are significantly positive (all at the 1% significance level) after controlling for other variables, indicating that enterprises’ digital transformation is positively correlated with the total number of patents acquired in each category (i.e., the higher an enterprise’s digital transformation, the higher its innovation potential). This result confirms hypothesis H1.

**Table 4:** Regression results of the reference model.

	(1)	(2)	(3)	(4)
	ln_patentscul	ln_inventioncul	ln_utilitymodelcul	ln_designcul
L.ln_digi2	0.1161***	0.0929***	0.0707***	0.3136***
	(8.89)	(5.82)	(3.63)	(9.96)
Size	0.6469***	0.6989***	0.5550***	0.3168***
	(40.76)	(34.28)	(20.59)	(6.89)
Lev	− 0.1140	− 0.5026***	0.0006	− 0.0853
	(− 1.15)	(− 4.21)	(0.00)	(− 0.34)
ROA	− 0.1011	− 0.6301**	0.0850	0.8636
	(− 0.45)	(− 2.33)	(0.25)	(1.53)
Top10	0.2528**	− 0.1167	− 0.0280	1.5767***
	(2.37)	(− 0.90)	(− 0.18)	(5.87)
Indep	0.3123	0.9579***	0.1082	0.3784
	(1.27)	(3.14)	(0.28)	(0.58)
Cashflow	0.8215***	0.2131	0.4145	1.0499*
	(3.35)	(0.72)	(1.16)	(1.67)
_cons	− 12.3803***	− 14.1906***	− 11.9490***	− 6.4778***
	(− 27.64)	(− 23.42)	(− 15.24)	(− 4.23)
Year	Yes	Yes	Yes	Yes
Industry	Yes	Yes	Yes	Yes
N	4294	3987	2821	1752
R^2^	0.544	0.433	0.458	0.256
F	60.5082	35.4997	28.5446	8.0279

The t-statistics are given in brackets.

*p < 0.1.

**p < 0.05.

***p < 0.01.

Second, the estimated coefficients of the independent variables represent the influence elasticity, reflecting in turn the degree of improvement in the cumulative total number of patents acquired by firms when digital transformation increases by 1%, since the main independent variables and the dependent variables in this study are treated with natural logarithms. Taking the cumulative total number of R&D patents acquired in column (1) as an example, the estimated coefficient of the main independent variable is 0.1161%, indicating that the cumulative total number of R&D patents acquired increases by 0.1161% for every 1% increase in digital transformation. This degree of influence is relatively significant considering that the number of R&D patents in this paper is a cumulative variable. Comparing the impact of the different types of patents, the cumulative number of design patents obtained has the largest impact, followed by the cumulative total number of R&D patents obtained, the cumulative number of invention patents obtained, and the cumulative number of utility model patents obtained. Invention patents tend to be less complex to develop and are therefore the easiest to modernize. R&D patents and utility model patents are more technically complex and often require a long technical accumulation process and repeated testing and review, so their impact is relatively slow. Nevertheless, the results of this paper show that the impact of digital transformation on the improvement of business capabilities is still highly significant. Additionally, at the R&D level, the economic impact of digital transformation on business innovation is present.

There are many factors that affect enterprise innovation capability; among the control variables considered in this paper, the effect of firm size on innovativeness is most evident, as shown in Table 4. The larger a firm is, the more R&D resource reserves and far-sighted strategic vision it has, and it can thus identify the direction of potential technology development in the industry as early as possible, organize researchers, and invest R&D funds in research and development. Small enterprises, on the other hand, have relatively weak human and material resources and strategic planning capacity, and are therefore, all other things being equal, unable to accumulate R&D results. In addition, sufficient cash flow and low debt levels can help to increase the innovation capability of enterprises.

### Robustness tests

In this paper, several methods are used to test the robustness of the baseline model. First, controlling for fixed effects; second, substituting independent variables; third, substituting the main independent variables; and fourth, using the instrumental variables approach to address possible endogeneity problems and revalidate the model in conjunction with the calibration of the main independent variables.

#### Controlling fixed effects

Individual differences between different companies are more obvious than other types of differences; for example, some companies have a strong innovation atmosphere and a good R&D environment, while others tend to acquire R&D patents through mergers and acquisitions and other external channels. These factors not only affect the amount of R&D patents held by companies, but are also difficult to fully quantify. At the same time, we control for time fixed effects, as different years can be affected by factors such as policies that change over time and are difficult to observe. Similarly, we also control for industry fixed effects, to account for differences in the degree of competition for R&D across industries, industry incentives, and other factors that are independent of individuals but are industry-specific. Moreover, as described in the model building section, the main independent variables in this paper are lagged by one period because of the lag in patent acquisition. The results in Table 4 show that the previous results of the study remain robust even after accounting for changes in these factors.

#### Replacing the window period

Considering the 2008 financial crisis and the 2018 US–China trade war, the three years before and after these two external environments appeared (i.e., 2007–2009 and 2017–2019) are excluded from the full sample and the benchmark regression is re-run. As shown in Tables 5 and 6, the regression results of each model after excluding some years are basically consistent with the benchmark regression; thus, the benchmark model can be considered robust.

**Table 5:** First robustness test.

	(1)	(2)	(3)	(4)
	ln_patentscul	ln_inventioncul	ln_utilitymodelcul	ln_designcul
L.ln_digi2	0.1139***	0.0902***	0.0734***	0.3131***
	(8.75)	(5.46)	(3.79)	(9.65)
Size	0.6476***	0.7030***	0.5482***	0.3136***
	(38.23)	(30.46)	(18.65)	(5.97)
Lev	− 0.1089	− 0.5194***	0.0375	− 0.0718
	(− 1.09)	(− 4.13)	(0.26)	(− 0.29)
ROA	− 0.0951	− 0.6197**	0.0554	0.8803
	(− 0.42)	(− 2.25)	(0.14)	(1.58)
Top10	0.2608**	− 0.1082	− 0.0175	1.5834***
	(2.33)	(− 0.78)	(− 0.11)	(5.66)
Indep	0.2692	0.9020***	0.0113	0.3811
	(1.12)	(3.00)	(0.03)	(0.60)
Cashflow	0.7776***	0.1661	0.4721	1.0146
	(3.00)	(0.54)	(1.25)	(1.49)
_cons	− 11.6350***	− 14.0878***	− 10.5313***	− 6.2489***
	(− 27.46)	(− 26.30)	(− 13.77)	(− 5.25)
Year	Yes	Yes	Yes	Yes
Industry	Yes	Yes	Yes	Yes
*N*	4292	3987	2821	1752
*R*^2^	0.540	0.431	0.453	0.254
F				

The t-statistics are given in brackets.

*p < 0.1.

**p < 0.05.

***p < 0.01.

**Table 6:** Second robustness test.

	(1)	(2)	(3)	(4)
	ln_patentscul	ln_inventioncul	ln_utilitymodelcul	ln_designcul
L.ln_digi2	0.1232***	0.1065***	0.0809***	0.3176***
	(8.18)	(5.57)	(3.53)	(8.45)
Size	0.6341***	0.6937***	0.5739***	0.2868***
	(32.67)	(26.31)	(17.01)	(4.84)
Lev	− 0.0446	− 0.4282***	− 0.0382	− 0.0072
	(− 0.40)	(− 3.06)	(− 0.23)	(− 0.02)
ROA	0.0628	− 0.7552**	0.0588	1.1866
	(0.22)	(− 2.07)	(0.12)	(1.54)
Top10	0.2171*	− 0.2184	− 0.0685	1.4721***
	(1.71)	(− 1.39)	(− 0.37)	(4.50)
Indep	0.5413*	0.8187**	0.4595	0.3704
	(1.89)	(2.36)	(1.06)	(0.48)
Cashflow	0.8131***	0.1806	0.3464	0.9706
	(2.74)	(0.53)	(0.80)	(1.31)
_cons	− 12.1503***	− 13.9337***	− 12.3923***	− 5.6198***
	(− 22.87)	(− 19.19)	(− 13.30)	(− 3.93)
Year	Yes	Yes	Yes	Yes
Industry	Yes	Yes	Yes	Yes
*N*	4292	3987	2821	1752
*R*^2^	0.547	0.435	0.458	0.249
F				

The t-statistics are given in brackets.

*p < 0.1.

**p < 0.05.

***p < 0.01.

#### Changing the measurement method of dependent variables

Since patent applications take a long time and have a certain time lag, this paper, following Chen, He and Zhang^90^, does not use the number of patents obtained in the current period as the independent variable, but rather the cumulative number of patents obtained in the base part of the regression, which allows us to better represent the impact of digital transformation on innovativeness over time. At the same time, by treating enterprises’ digital transformation with a lag of one period, we can to some extent eliminate the endogenous disturbances caused by possible reverse causality and reflect the time lag. However, the aggregate of acquired patents is not necessarily the result of an enterprise’s own R&D. There may be a small portion of patents acquired through mergers and acquisitions of other companies, and this portion of innovation capability may be independent of the topic of the digital transformation studied in this paper. To avoid this possible source of confounding, this paper, referring to Hall and Harhoff^18^, replaces the original dependent variables with the cumulative number of patents filed by firms and reruns the test. The results presented in Table 7 show that the estimated coefficients of the main independent variables in columns (1) to (4) are significantly positive (in descending order, at the 1%, 1%, 1% and 5% significance levels), indicating a significant upward effect of digital transformation on the cumulative number of R&D patents filed (i.e., the greater the enterprises’ digital transformation, the greater) their innovation potential. These findings confirm the relative robustness of the control results.

**Table 7:** Third robustness test.

	(1)	(2)	(3)	(4)
	ln_patents	ln_invention	ln_utilitymodel	ln_design
L.ln_digi2	0.1224***	0.1486***	0.0193	0.1095**
	(7.22)	(7.28)	(0.69)	(2.23)
Size	0.6881***	0.7099***	0.5594***	0.2785***
	(37.69)	(30.93)	(15.37)	(4.26)
Lev	− 0.0785	0.0103	0.3761**	0.6229*
	(− 0.70)	(0.07)	(1.96)	(1.81)
ROA	0.4315	1.4345***	0.3348	0.2485
	(1.53)	(3.94)	(0.67)	(0.32)
Top10	0.5667***	0.2808*	0.6098***	1.9447***
	(4.54)	(1.85)	(2.85)	(4.82)
Indep	0.3967	0.8293**	− 0.0632	0.4030
	(1.26)	(2.25)	(− 0.12)	(0.39)
Cashflow	1.1847***	1.1635***	0.7652	− 0.1087
	(4.12)	(3.37)	(1.62)	(− 0.13)
_cons	− 13.8686***	− 14.5661***	− 13.5911***	− 6.5329***
	(− 28.84)	(− 24.78)	(− 13.60)	(− 3.81)
Year	Yes	Yes	Yes	Yes
Industry	Yes	Yes	Yes	Yes
N	3887	3145	1600	632
R^2^	0.497	0.445	0.352	0.270
F	44.6911	30.2759	11.3489	3.3309

*p < 0.1.

**p < 0.05.

***p < 0.01.

#### Changing the measurement method of the core independent variables

In this paper, following Xiao, Sun, Yuan and Sun^91^, Huang, Xie, Meng and Zhang^14^, and Wu, Chang and Ren^4^, different terms are used to measure the enterprises’ digital transformation, excluding the term “application of digital technology” at the application level and keeping only “artificial intelligence technology” at the basic digital technology level. “Blockchain technology”, “cloud computing technology,” and “big data technology” are retained at the basic digital technology level, and the natural logarithm of their commonality is used as a surrogate variable to test robustness. According to the regression results presented in Table 8, the estimated coefficients of the main independent variables in each column of the results remain significantly positive (all at the 1% significance level) when different measures of digital transformation are used. Furthermore, comparing the elasticity coefficients of the impact of digital transformation on the total volume of patents acquired, design patents continue to have the largest impact, followed by aggregate R&D patents, invention patents and utility model patents. These findings are consistent with the benchmark regression results.

**Table 8:** Fourth robustness test.

	(1)	(2)	(3)	(4)
	ln_patentscul	ln_inventioncul	ln_utilitymodelcul	ln_designcul
L.ln_digi2	0.1304***	0.0956***	0.0907***	0.2861***
	(9.06)	(5.34)	(4.19)	(8.32)
Size	0.6502***	0.7037***	0.5556***	0.3359***
	(41.15)	(34.66)	(20.70)	(7.27)
Lev	− 0.1043	− 0.5137***	0.0015	− 0.0912
	(− 1.06)	(− 4.31)	(0.01)	(− 0.37)
ROA	− 0.1118	− 0.6644**	0.0775	0.7479
	(− 0.50)	(− 2.46)	(0.23)	(1.31)
Top10	0.2832***	− 0.0968	− 0.0069	1.6302***
	(2.66)	(− 0.74)	(− 0.04)	(6.01)
Indep	0.2569	0.9106***	0.0377	0.2806
	(1.04)	(2.98)	(0.10)	(0.43)
Cashflow	0.8046***	0.2022	0.4285	0.9629
	(3.29)	(0.68)	(1.20)	(1.52)
_cons	− 12.4076***	− 14.2548***	− 11.9192***	− 6.6978***
	(− 27.74)	(− 23.53)	(− 15.22)	(− 4.33)
Year	Yes	Yes	Yes	Yes
Industry	Yes	Yes	Yes	Yes
N	4292	3987	2821	1752
R^2^	0.544	0.432	0.459	0.243
F	60.5868	35.3894	28.6434	7.4997

The t-statistics are given in brackets.

*p < 0.1.

**p < 0.05.

***p < 0.01.

#### Endogeneity test

Given the possible endogeneity of the relationship between digital transformation and enterprises’ innovation capability, the digital transformation variable in the base section of the regression is shifted by one period to avoid a violation of endogeneity due to possible reverse causality. At the same time, this paper continues to search for instrumental variables, building on a related study by Fishman and Svensson^21^ in which the industry average of digital transformation is used as an instrumental variable for the least squares estimation (2SLS), in order to observe the net effect of digital transformation on innovation capability.

As shown in Table 9, the estimated coefficients of the main independent variables for digital transformation in the results in columns (1), (2), and (3) are 0.0965, 0.0861, and 0.1329, respectively, and all results are significantly positive. This indicates that, after excluding possible endogenous disturbances associated with bidirectional causality, the enterprises’ digital transformation still has a significant upward effect on innovativeness. In the regression results in column (4), the estimated coefficient of the main independent variable—digital transformation—is still positive but is not significant, suggesting that possible bilateral causality overestimates the impact of digital transformation on firms’ design patents. This also suggests that, for transforming firms represented by listed A-shares, digital transformation technology still mainly affects the output of patents with technological content (i.e. it mainly improves innovation capability when there is a higher level of technological content).

**Table 9:** Endogeneity test.

	(1)	(2)	(3)	(4)
	ln_patentscul	ln_inventioncul	ln_utilitymodelcul	ln_design
L.ln_digi2	0.0965***	0.0861***	0.1329***	0.0369
	(4.89)	(3.92)	(4.36)	(0.46)
Size	0.5429***	0.6099***	0.3793***	0.1782***
	(31.26)	(29.70)	(12.40)	(2.82)
Lev	0.0651	− 0.5645***	0.3366**	0.7919**
	(0.57)	(− 4.50)	(2.01)	(2.38)
ROA	0.0712	− 0.1227	− 0.0440	0.1282
	(0.27)	(− 0.43)	(-0.11)	(0.16)
Top10	0.4285***	− 0.0808	0.2716	1.9361***
	(3.50)	(− 0.59)	(1.45)	(4.84)
Indep	0.5183*	1.0240***	0.6246	0.3165
	(1.84)	(3.21)	(1.35)	(0.30)
Cashflow	0.4052	0.0127	0.6398	0.7018
	(1.44)	(0.04)	(1.52)	(0.84)
_cons	− 8.9874***	− 11.2535***	− 6.8570***	− 3.1225**
	(− 20.75)	(− 19.21)	(− 8.28)	(− 2.26)
Year	Yes	Yes	Yes	Yes
N	4292	3987	2821	632
R^2^	0.347	0.322	0.164	0.105
F	110.1710	92.4409	26.6337	3.4879

The t-statistics are given in brackets.

*p < 0.1.

**p < 0.05.

***p < 0.01.

In summary, this paper considers different sources of potential confounding factors in the research process and applies different approaches to target processing. The results confirm the robustness of the empirical findings of this paper, as well as the reliability of the theoretical analysis. Building on the above robust results, this paper conducts a further analysis on this basis.

## Further research

### Mechanisms analysis

In the theoretical mechanism analysis section, this paper hypothesizes that digital transformation can improve enterprises’ innovation capability in two ways: by reducing agency costs and by reducing enterprises’ risk-bearing levels. This study uses the stepwise regression method put forth by Wen and Ye^92^ to test these two mechanisms. Following the work of Singh, Davidson and Suchard^86^, Xiao and Chen^87^, and Xu and Zhang^1^, total asset turnover (ATO) is selected as a proxy variable to measure the value of the trustworthy representation of enterprises. The construction of corporate risk (Risk) follows the practice of Yu, Li and Pan^88^ and Song, Wen, Wang and Shen^63^, using the volatility of corporate profits to represent risk.

The results of the regression test for the agency cost mechanism are presented in column (1) and column (2) of Table 10. The estimated coefficient of the main independent variable in column (1) is 0.0327, which is a significant positive value at the 1% significance level, indicating that there is a significant positive relationship between digital transformation and total corporate asset turnover. Digital transformation can increase the total asset turnover of the firm (i.e. reduce the enterprises’ agency costs). The estimated ATO coefficient of total asset turnover in column (2) is 0.2587 and is significantly positive at the 1% significance level, indicating that, the lower the agency cost, the higher the level of innovativeness. The regression results confirm the agency cost mediation test. That is, the higher the enterprise’s digital transformation, the higher the turnover rate and the lower the agency costs, which ultimately increases the enterprises innovativeness, confirming hypothesis H2a.

**Table 10:** Test for mediating effect.

	(1)	(2)	(3)	(4)
	ATO	ln_patentscul	Risk	ln_patentscul
ln_digi2	0.0327***		2.6374***	
	(10.98)		(3.71)	
L.ln_digi2		0.1124***		0.1105***
		(8.64)		(6.56)
ATO		0.2587***		
		(6.74)		
Risk				0.0057***
				(4.81)
Size	0.0220***	0.6536***	− 2.8770***	0.6653***
	(8.79)	(41.32)	(− 4.91)	(29.96)
Lev	0.0069***	− 0.2401***	63.9903***	− 0.2370**
	(3.30)	(− 2.40)	(112.77)	(− 1.87)
ROA	0.1311***	− 0.4151	127.6630***	0.3422
	(8.45)	(− 1.82)	(72.04)	(1.09)
Top10	0.1655***	0.2181**	6.3565	0.2188
	(8.25)	(2.06)	(1.37)	(1.53)
Indep	− 0.1903***	0.3811	3.4167	0.6145**
	(− 3.67)	(1.56)	(0.28)	(2.08)
Cashflow	0.4601***	0.6514***	42.9052***	0.8487***
	(13.52)	(2.66)	(5.44)	(2.74)
_cons	0.1153*	− 12.7008***	36.8423**	− 12.8449***
	(1.72)	(− 28.34)	(2.36)	(− 21.67)
Year	Yes	Yes	Yes	Yes
Industry	Yes	Yes	Yes	Yes
N	31,789	4292	24,582	2881
R^2^	0.244	0.549	0.362	0.537
F	103.2852	60.9591	144.4438	

The t-statistics are given in brackets.

*p < 0.1.

**p < 0.05.

***p < 0.01.

The regression results used to test the risk level mechanism are shown in column (3) and column (4) of Table 10. The estimated coefficient of the main independent variable in column (3) is 2.6374, which is significantly positive at the 1% significance level, indicating that digital transformation significantly increases the risk-bearing levels of enterprises. The estimated coefficient of the variable risk in column (4) is 0.0057, which is a significant positive value at the 1% significance level. Hence, digital transformation can increase the innovation capability of enterprises by enhancing their risk-bearing levels. That is, the greater the digital transformation, the higher the volatility of an enterprise’s profitability, thus increasing the enterprise’s risk-bearing level and ultimately enhancing its innovation capability. The risk-bearing level mechanism developed in hypothesis H2b is thus confirmed.

### Heterogeneity analysis

The relationship between digital transformation and an enterprise’s innovation capability may vary depending on the type of enterprise. Therefore, this paper compares the regression results of the SME and GEM sample with the full benchmark sample in order to better clarify the characteristics of the role that digital transformation plays, and to provide an evidence base for further optimizing the digital management of companies. Table 11 shows the regressions for all A-share companies in columns (1) and (2), the regression results for the GEM sample in columns (3) and (4), and the regression results for the SME version in columns (5) and (6). The following conclusions are drawn from the comparative analysis.

**Table 11:** Heterogeneity test.

	(all)	(all)	(GEM)	(4)	(SMEs)	(6)
	ln_patentscul	ln_Invention	ln_patentscul	ln_Invention	ln_patentscul	ln_Invention
L.ln_digi2	0.1161***	0.1486***	0.1113***	0.1170***	0.1684***	0.0798**
	(8.89)	(7.28)	(5.94)	(3.16)	(7.25)	(2.15)
Size	0.6469***	0.7099***	0.5219***	0.7427***	0.6058***	0.7455***
	(40.76)	(30.93)	(15.39)	(12.04)	(18.94)	(13.39)
Lev	− 0.1140	0.0103	0.3333**	− 0.3379	− 0.3345*	0.3283
	(− 1.15)	(0.07)	(2.09)	(− 1.10)	(− 1.94)	(1.17)
ROA	− 0.1011	1.4345***	− 0.3424	1.4783**	0.3480	1.5599**
	(− 0.45)	(3.94)	(− 1.09)	(2.10)	(0.90)	(2.39)
Top10	0.2528**	0.2808*	0.4748**	− 0.1752	0.0889	− 0.0261
	(2.37)	(1.85)	(2.46)	(− 0.48)	(0.51)	(− 0.09)
Indep	0.3123	0.8293**	0.3017	− 0.3778	− 0.9181**	− 0.3620
	(1.27)	(2.25)	(0.73)	(− 0.44)	(− 2.09)	(− 0.50)
Cashflow	0.8215***	1.1635***	0.2349	0.3292	1.2193***	1.9816***
	(3.35)	(3.37)	(0.60)	(0.44)	(2.97)	(3.14)
_cons	− 12.3803***	− 14.5661***	− 8.6843***	− 14.8746***	− 11.3379***	− 14.5152***
	(− 27.64)	(− 24.78)	(− 10.78)	(− 10.85)	(− 12.25)	(− 8.71)
Year	Yes	Yes	Yes	Yes	Yes	Yes
Industry	Yes	Yes	Yes	Yes	Yes	Yes
*N*	4292	3145	1336	619	1531	1059
*R*^2^	0.544	0.445	0.484	0.422	0.538	0.402
F	60.5082	30.2759	22.7133	8.8732	24.6190	10.6126

	(all)	(all)	(GEM)	(GEM)	(SMEs)	(SMEs)
	ln_patentscul	ln_invention	ln_patentscul	ln_invention	ln_patentscul	ln_invention
L.ln_digi2	0.1161***	0.1486***	0.1113***	0.1170***	0.1684***	0.0798**
	(8.89)	(7.28)	(5.94)	(3.16)	(7.25)	(2.15)
Size	0.6469***	0.7099***	0.5219***	0.7427***	0.6058***	0.7455***
	(40.76)	(30.93)	(15.39)	(12.04)	(18.94)	(13.39)
Lev	− 0.1140	0.0103	0.3333**	− 0.3379	− 0.3345*	0.3283
	(− 1.15)	(0.07)	(2.09)	(− 1.10)	(− 1.94)	(1.17)
ROA	− 0.1011	1.4345***	− 0.3424	1.4783**	0.3480	1.5599**
	(− 0.45)	(3.94)	(− 1.09)	(2.10)	(0.90)	(2.39)
Top10	0.2528**	0.2808*	0.4748**	− 0.1752	0.0889	− 0.0261
	(2.37)	(1.85)	(2.46)	(− 0.48)	(0.51)	(− 0.09)
Indep	0.3123	0.8293**	0.3017	− 0.3778	− 0.9181**	− 0.3620
	(1.27)	(2.25)	(0.73)	(− 0.44)	(− 2.09)	(− 0.50)
Cashflow	0.8215***	1.1635***	0.2349	0.3292	1.2193***	1.9816***
	(3.35)	(3.37)	(0.60)	(0.44)	(2.97)	(3.14)
_cons	− 12.3803***	− 14.5661***	− 8.6843***	− 14.8746***	− 11.3379***	− 14.5152***
	(− 27.64)	(− 24.78)	(− 10.78)	(− 10.85)	(− 12.25)	(− 8.71)
Year	Yes	Yes	Yes	Yes	Yes	Yes
Industry	Yes	Yes	Yes	Yes	Yes	Yes
N	4292	3145	1336	619	1531	1059
R^2^	0.544	0.445	0.484	0.422	0.538	0.402
F	60.5082	30.2759	22.7133	8.8732	24.6190	10.6126

The t-statistics are given in brackets.

*p < 0.1.

**p < 0.05.

***p < 0.01.

First, digital transformation has a significant impact on the innovativeness of listed firms in different sectors, with the estimated coefficients of the main independent variables in the respective columns of the results all being significantly positive. The impact on the total number of R&D patents and invention patents is even more significant, demonstrating the universality of the effect of digital transformation.

Second, due to the fact that the main independent variables in this study are transformed into natural logarithms, the estimated coefficients of the independent variables are influence elasticities, which in turn allow us to compare the strength of the influence of each group. If we take patents on inventions as an observational benchmark, the influence of digital transformation on the innovation capability of GEM firms is higher than that of SMEs (0.1170% versus 0.0798%), mainly because GEM firms generally have clear technological advantages, both in terms of their stock of technological resources and their experience in R&D management in general, which are better than those of SMEs. Therefore, controlling for other influencing factors, the same digital transformation has a stronger effect on the innovation capability of GEM firms. Second, the impact of digital transformation on innovation capability is lower in both the GEM and SME board samples than in the overall sample, implying that the greater benefit of digital transformation still accrues to the larger companies in the main board.

### Analysis of the moderation effects

The empirical results obtained in the previous section confirm the impact of digital transformation on enterprises’ innovation capabilities, but the specific mechanism at work here requires further analysis. According to the theoretical analysis in the previous section, the degree of influence of digital transformation on enterprises’ innovativeness may vary according to the change in certain internal and external factors (e.g. internal factors such as enterprise characteristics and degree of openness and external factors such as institutional investors’ participation ratio in enterprise capital and industry concentration). Identifying the overlapping influence of these factors is useful in terms of optimizing enterprise management and maximizing enterprises’ innovativeness. The results of the regression test of the moderating effect are shown in Table 12. The estimated coefficients of the main independent variables and the cross-sectional multipliers in column (1) are significantly positive at the 1% significance level, indicating that, when controlling for other influencing factors, digital transformation can induce SOEs to perform more R&D than private enterprises at the same intensity. The impact of digital transformation on the innovation capability of SOEs is stronger than that of private enterprises, which supports hypothesis H3a.

**Table 12:** Test of modifying effects.

	(1)	(2)	(3)	(4)
	ln_patentscul	ln_patentscul	ln_patentscul	ln_patentscul
L.ln_digi2	0.1014***	0.0935***	0.0940***	0.1486***
	(7.34)	(5.20)	(6.77)	(9.55)
L.ln_digi2_SOE	0.0911***			
	(3.59)			
SOE	− 0.0587			
	(− 1.25)			
Size	0.6403***	0.6387***	0.5477***	0.5433***
	(39.51)	(38.73)	(32.05)	(28.46)
Lev	− 0.1337	− 0.1289	0.0570	0.1150
	(− 1.35)	(− 1.30)	(0.51)	(1.04)
ROA	− 0.1589	− 0.1303	0.0916	0.0383
	(− 0.71)	(− 0.58)	(0.36)	(0.15)
Top10	0.2533**	0.1995*	0.3885***	0.4783***
	(2.38)	(1.71)	(3.23)	(3.74)
Indep	0.3066	0.3478	0.5223*	0.4977*
	(1.25)	(1.41)	(1.87)	(1.83)
Cashflow	0.8470***	0.8116***	0.2154	0.4718
	(3.46)	(3.31)	(0.78)	(1.62)
L.ln_digi2_INST		0.0730*		
		(1.88)		
INST		0.0118		
		(0.14)		
L.ln_digi2_Overinc			0.1805***	
			(3.36)	
Overinc			0.5575***	
			(5.15)	
L.ln_digi2_HHI				− 0.3781***
				(− 3.93)
HHI				− 0.0735
				(− 0.36)
_cons	− 12.2236***	− 12.1789***	− 9.1371***	− 9.0334***
	(− 27.04)	(− 26.48)	(− 21.31)	(− 17.52)
Year	Yes	Yes	Yes	Yes
Industry	Yes	Yes	Yes	Yes
N	4292	4292	4292	4290
R^2^	0.546	0.545	0.365	0.351
F	59.4234	59.1898	111.6441	89.1892

The t-statistics are given in brackets.

*p < 0.1.

**p < 0.05.

***p < 0.01.

Column (2) of Table 12 shows that the estimated coefficients of the core explanatory variables and the cross-multiplier terms are significantly positive at the 1% significance level. This suggests that institutional investor shareholding positively moderates the relationship between digital transformation intensity and enterprises’ innovation capability (i.e., the higher the institutional investor shareholding, the stronger the enterprises’ innovation capability). When the shareholding ratio of institutional investors is higher, not only will the institution itself pay more attention to the daily production and operation of the enterprise, but these circumstances will also help the enterprise gain the attention of other institutions and retail investors through publishing research reports and performance forecasts. This external attention creates moderate pressure on the enterprises to further improve their performance by strengthening their management, thus reinforcing the role of digital transformation in enhancing innovation capacity, proving hypothesis H3b.

Column (3) of Table 12 shows that the estimated coefficients of the core explanatory variables and the cross-multiplier terms are all significantly positive. In this paper, the openness level of enterprises is measured by the proportion of enterprises’ overseas business revenue. A higher level of overseas business revenue implies a higher degree of enterprises’ openness to the outside world, indicating that the degree of openness to the outside world positively moderates the relationship between the intensity of digital transformation and enterprises’ innovation ability. Through internationalization, enterprises can produce a reverse technology spillover effect on the one hand and borrow advanced management concepts from abroad on the other hand, which in turn can strengthen the effect of digital transformation on the enhancement of the enterprise’s innovation ability. Hypothesis H3c is thus verified.

As shown in column (4) of Table 12, the estimated coefficients of the core explanatory variables and the cross-multiplier terms are opposing (i.e., the higher the industry concentration, the lower the effect of digital transformation on the enhancement of enterprises’ innovation capabilities, all other influencing factors being consistent). This paper uses the Herfindahl Index (HHI) to measure industry concentration. The larger the HHI value, the more monopolized the industry in which the enterprise is located. For industries with strong monopoly power, the lack of competition will lead to insufficient incentives for enterprises to reform and innovate, and thus the effect of digital transformation is greatly reduced. Therefore, the conclusions of this paper are consistent with hypothesis H3d, which states that reducing the monopoly power of the industry and improving the level of competition will help to fully absorb the effect of digital transformation on the enhancement of the innovation ability of enterprises.

## Conclusion and discussion

### Research conclusion

This paper investigates the mechanism and impact of digital transformation on enterprises’ innovation capability using the data of Chinese listed A-share enterprises from 2007 to 2021. The results of the study show that, first, digital transformation has a positive effect on enterprises’ innovation capability. The results of the data regression show that more intensive digital transformation can motivate an enterprise to achieve greater innovation output. In this paper, benchmarking regressions on innovation capability using groups of variables enriches the variable setting of existing studies, as well as the theoretical mechanisms. At the same time, digital transformation can help companies to break through, innovate, and re-innovate in today’s digital economy, thereby improving their core competitiveness and strengthening their position in the digital economy market. Second, agency costs and the extent of risk-bearing level mediate the relationship between digital transformation and enterprises’ innovation capability.

### Theoretical significance

Most scholars use innovation application^93,94^ and R&D input ratio^95^ as proxy variables to measure corporate innovation, but digital transformation is a dynamic process that creates a complex environment. In this paper, we expand existing research to encompass cumulative innovation application, which reflects dynamic capability theory. We constructed a form of digital transformation using text mining technology to replace the dummy variable used in other papers. The conclusions drawn in this study creatively complement and extend the mechanisms of the impact of digital transformation on enterprises’ innovative capabilities. On the one hand, digital transformation lowers the costs of an enterprise’s headmaster agent while accounting for the fact that lowering the costs of an enterprise’s headmaster agent can increase its innovativeness. On the other hand, this paper argues that digital transformation can effectively reduce the volatility of corporate profits in order to reduce risk-bearing level; doing so provides more opportunities to help companies improve their innovation capabilities. By linking the influence mechanisms of digital transformation and corporate innovation capability to agent costs and risk-bearing levels, respectively, this paper creatively proposes and demonstrates the mediating role of agency costs and risk-bearing level. Third, the impact of digital transformation on the innovation capability of enterprises of different natures and sectors is heterogeneous, which has strong theoretical guiding significance. In particular, digital transformation has a stronger impact on the innovation potential of SOEs than on that of private enterprises. Additionally, an increase in the share of shares owned by institutional investors, a greater opening of companies to the outside world, and a reduction in industry concentration can all increase the impact of digital transformation on the innovation capabilities of enterprises. This analysis shows that, digital transformation has a general impact on the innovativeness of listed companies across all sectors. Further analysis shows that digital transformation has a stronger impact on the innovative capabilities of large companies than on that of small and medium-sized companies. Many advantages of large listed companies, such as a sufficient capital chain and the ability to manage resources, mean that large listed companies are able to benefit more from digital transformation.

### Practical implications

Based on the above findings, this chapter makes the following recommendations.

At the enterprise level, corporate managers should appropriately lead their organizations in making and implementing sustained and beneficial decisions for long-term digital transformation, with a focus on the impact of big data on the enterprise’s digital infrastructure and development. First, the use of digital technologies by enterprises can change the basic form and function of their products, increase product acceptance, and achieve the basic goals of product innovation. Second, digitalization can change the internal governance structure of enterprises, focusing on the strategic changes of enterprises at different stages of transformation. Enterprises should make full use of integrated and shared digital resources to improve the efficiency of traditional configuration, optimize the internal management structure, and explore effective ways to reduce the cost of the entrusted agency of the enterprise and improve the enterprise’s risk-bearing, so as to realize the sustainable and maximum release of the value of the enterprise’s digital transformation. Finally, enterprises should take digital transformation as a way to make breakthroughs in innovation, accelerate the flow of resources between internal and external enterprises, and promote the realization of internal and external collaborative innovation. Enterprises must be brave enough to bear the various risks and challenges on the road of transformation, so as to promote the realization of high-quality development.

At the government level, the government should actively create a digital environment and provide policy support. Meanwhile, based on the discussion in the heterogeneity section of this paper, the digital transformation of enterprises in different industries has different degrees of impact on innovation capability. The government should introduce special policies for enterprises in different industries and regions. In this way, the government can assist enterprises in establishing public digital platforms, create a better open environment in which enterprises can implement digitalization, and lower their digitalization thresholds. Through the flexible application of financial subsidies, tax exemptions, and other targeted policies, the government can support some enterprises to carry out digital transformation and reduce the monopoly present in the industry. Relevant government departments should also improve the supporting regulatory and governance systems for digital transformation, in order to further standardize the development of the digital economy.

### Research limitations

As the research samples are from Chinese enterprises among different types of industries, the findings of this study cannot necessarily be generalized to other countries. Additionally, our understanding of the mechanism at work in regard to digital transformation and corporate innovation still needs to be enriched in the future.

## Data Availability

The data that support the findings of this study are available from the corresponding author upon reasonable request.
